# Remodeling of Stromal Immune Microenvironment by Urolithin A Improves Survival with Immune Checkpoint Blockade in Pancreatic Cancer

**DOI:** 10.1158/2767-9764.CRC-22-0329

**Published:** 2023-07-12

**Authors:** Siddharth Mehra, Vanessa T. Garrido, Austin R. Dosch, Purushottam Lamichhane, Supriya Srinivasan, Samara P. Singh, Zhiqun Zhou, Iago De Castro Silva, Chandrashekar Joshi, Yuguang Ban, Jashodeep Datta, Eli Gilboa, Nipun B. Merchant, Nagaraj S. Nagathihalli

**Affiliations:** 1 Department of Surgery, University of Miami, Miller School of Medicine, Miami, Florida.; 2 Department of Biochemistry, Mangalore University, Karnataka, India.; 3 Department of Biostatistics and Bioinformatics, University of Miami, Miami, Florida.; 4 Sylvester Comprehensive Cancer Center, University of Miami, Miami, Florida.; 5 Department of Microbiology and Immunology, University of Miami, Miami, Florida.

## Abstract

Pancreatic ductal adenocarcinoma (PDAC) is a significant contributor to cancer-related morbidity and mortality, and it is known for its resistance to conventional treatment regimens, including chemotherapy and immune checkpoint blockade (ICB)-based therapies. We have previously shown that Urolithin A (Uro A), a gut microbial metabolite derived from pomegranates, can target and inhibit *KRAS*-dependent PI3K/AKT/mTOR signaling pathways to overcome therapeutic resistance and improve survival in PDAC. However, the effect of Uro A on the tumor immune microenvironment and its ability to enhance ICB efficacy has not been explored. This study demonstrates that Uro A treatment reduces stromal fibrosis and reinvigorates the adaptive T-cell immune response to overcome resistance to PD-1 blockade in a genetically engineered mouse model (GEMM) of PDAC. Flow cytometric–based analysis of Uro A-treated mouse tumors revealed a significant attenuation of immunosuppressive tumor-associated M2-like macrophages with a concurrent increase in the infiltration of CD4^+^ and CD8^+^ T cells with memory-like phenotype along with reduced expression of the exhaustion-associated protein, PD-1. Importantly, the combination of Uro A treatment with anti-PD-1 immunotherapy promoted enhancement of the antitumor response with increased infiltration of CD4^+^ Th1 cells, ultimately resulting in a remarkable improvement in overall survival in GEMM of PDAC. Overall, our findings provide preclinical evidence for the potential of Uro A as a novel therapeutic agent to increase sensitivity to immunotherapy in PDAC and warrant further mechanistic exploration in preclinical and clinical studies.

**Significance:**

Immunotherapeutic agents are ineffective against pancreatic cancer, mainly due to the immunosuppressive tumor microenvironment and stromal desmoplasia. Our current study demonstrates the therapeutic utility of a novel gut microbial metabolite, Uro A, to remodel the stromal-immune microenvironment and improve overall survival with anti-PD-1 therapy in pancreatic cancer.

## Introduction

Pancreatic ductal adenocarcinoma (PDAC) is a highly aggressive cancer with a 5-year survival rate of 12%, making it one of the most lethal solid organ malignancies (1). Treatment options for PDAC remain limited, with curative surgical resection possible in only 15%–20% of cases due to advanced disease at the time of diagnosis (1, 2). The current standard-of-care chemotherapy provides limited benefit in improving disease outcomes in most patients, yielding significant treatment-associated toxicities and frequently decreasing quality of life (3). Although immunotherapy has revolutionized the treatment of many malignancies, it has largely been ineffective in achieving durable responses in the majority of patients with PDAC (4–6). Studies aimed at elucidating the defining characteristics that render PDAC refractory to conventional chemotherapeutic and immunotherapeutic approaches have revealed that the tumor microenvironment (TME) serves as a crucial mechanism by which cancer cells evade immune surveillance, enabling tumor progression and distant metastatic spread (7). The PDAC TME is characterized by intense stromal desmoplasia, an abundance of extracellular matrix proteins, immunosuppressive cellular populations, and a striking absence of activated T cells. Previous studies have shown this resistant phenotype is reliant on oncogenic *KRAS* mutations to drive disease progression (8, 9).

Among the pleiotropic downstream drivers of *KRAS*-driven oncogenesis, multiple studies have demonstrated the importance of the PI3K/AKT/mTOR cascade in promoting the immunosuppressive properties of PDAC lesions via effects on both tumor cells and other immune constituents within the TME (10). Inactivation of key components of this signaling node has been shown to augment adaptive immune response and attenuate disease progression in PDAC (11).

Despite promising preclinical results demonstrating the therapeutic efficacy of this signaling axis, one major barrier to the clinical implementation of PI3K/AKT/mTOR pathway inhibitors for the treatment of PDAC is the narrow therapeutic window for targeting these proteins and the significant side effects associated with the treatment (12). Previous studies by our group have demonstrated that the natural compound Urolithin A (Uro A), a gut microbiome natural compound derived from pomegranates, potently inhibits PI3K/AKT/mTOR signaling at a physiologic dose that is well tolerated in mice (10, 13, 14). We have shown that Uro A exhibits strong antitumor effects combined with gemcitabine chemotherapy, resulting in reduced tumor growth and improved survival in the aggressive *Ptf1a^Cre/+^;LSL-Kras^G12D/+^;Tgfbr2^flox/flox^* (PKT) murine model of PDAC (10).

Despite these encouraging preclinical results, the impact of Uro A on stromal and immune architecture in the PDAC TME remains unknown. Our current study demonstrates that Uro A treatment can effectively reduce primary tumor burden in a T cell–dependent manner, decrease immunosuppressive tumor-associated macrophages (TAM), and significantly increase the infiltration of CD4^+^ and CD8^+^ T cells with a memory-like phenotype in the TME. Furthermore, we show combining Uro A and anti-PD-1 (aPD-1) therapy leads to a robust antitumor response, significantly improves overall survival, and promotes the expansion of effector CD4^+^ Th1 cells in the highly aggressive, syngeneic model of PDAC. Taken together, this study provides promising evidence that Uro A holds potential as a novel therapeutic option for the treatment of PDAC and could be further explored in clinical trials in combination with immune checkpoint blockade (ICB) in patients with this deadly malignancy.

## Materials and Methods

### *Ptf1a^Cre/+^;LSL-Kras^G12D/+^;Tgfbr2^flox/flox^* (PKT) Mouse Studies

Tumor-bearing PKT mice (provided by Dr. Harold Moses, Vanderbilt University Medical Center, Nashville, TN) were generated as described previously (10, 15, 16). Male and female PKT mice (4.5 weeks old) were used in this study and were housed in pathogen-free conditions under a 12-hour light-dark diurnal cycle with a controlled temperature of (21°C–23°C) and maintained on standard rodent diet.

PKT mice reproducibly develop highly aggressive PDAC tumors that result in mortality within 8 weeks if left untreated (17, 18). For endpoint-based analysis, PKT mice were treated with Uro A (20 mg/kg/mouse via oral gavage daily for 5 days per week) or equal volume vehicle control (10% glucose in water) beginning at 4.5 weeks of age for a total of 3 weeks before harvesting pancreatic tumors. Similarly, endpoint analysis was also conducted in PKT genetically engineered mouse models (GEMM) with Omipalisib, PI3K inhibitor (PI3Ki, GSK2126458). The drug was prepared in solvent containing 10% DMSO, 40% PEG-300, 5% Tween-80, and 45% saline. Beginning at 4.5 weeks of age, PKT mice were treated at 0.3 mg/kg/mouse via oral gavage for 5 days per week or equal volume of vehicle control. At the end of 2 weeks treatment, mice were euthanized and pancreatic tumor tissues were harvested for downstream experiments. For survival studies, PKT mice began treatment with vehicle, Uro A, αPD-1 antibody (BioXCell, Clone #BE0273, 200 μg/mouse, intraperitoneal injection twice weekly), or a combination of Uro A and αPD-1 at 4.5 weeks that continued until moribund. For αPD-1 treatment, antibody dosing was changed to once weekly when mice reached 7.5 weeks of age. Mice were also weighed biweekly to monitor for changes in their body weights due to treatment-associated toxicity. All PKT mice as part of our long-term treatment survival arm were monitored daily for their overall performance and status in a treatment-blinded fashion similar along the lines of previously published studies (10, 18, 19). Animals showing clinical signs of significant disease burden (impaired mobility, ruffled hair, hunched posture, loss of coordination) were euthanized.

### Histologic Analysis

Pancreatic tumor tissues were fixed in 10% neutral buffered formalin, embedded in paraffin, and sectioned. Hematoxylin and eosin (H&E), Sirius Red, Masson's trichrome, and Alcian Blue were performed as described previously (15, 20). For IHC, tissue sections were mounted on glass slides and deparaffinized in xylene, followed by rehydration using alcohol gradient. Antigen retrieval was performed by incubating samples in citrate buffer (0.01 mol/L, pH 6.0) and heating. Sections were blocked using BlockAid (Thermo Fisher Scientific) to preclude nonspecific binding. Endogenous peroxidase activity was quenched by incubating with 3% H_2_O_2_ for 10 minutes. Sections were then incubated with the primary antibodies listed in (Supplementary Table S1) in a humidified chamber at 4℃ overnight. The following day, slides were washed and developed using VECTASTAIN® Elite® ABC-HRP Kit, Peroxidase (Standard)-based kit (Vector) as per the manufacturer's protocol, with diaminobenzidine as the chromogen. Tissue sections were counterstained with Mayer's hematoxylin, mounted, and imaged using DM750 Leica microscope (Leica Microsystems). For the quantitative histologic analysis, we analyzed images obtained from pancreatic tumor tissue sections by focusing solely on the fraction of the tumor area in both the vehicle and treatment groups. To quantify the number of positive cells, we captured 3–4 fields of view within the tumor area in a treatment-blinded manner using ImageJ. To analyze the percentage of the tumor area in relation to the entire pancreas, we captured multiple fields of view (3–4) at lower magnification. We quantitatively compared total area of normal pancreatic architecture (without Pancreatic Intraepithelial Neoplasia (PanINs) and tumor ductal epithelial cells) with areas with tumor cells in each tissue section.

### Western Blot Analysis

Cell lysis and Western blotting were done as described previously (10). For *in vivo* studies*,* tumors were procured, and 5–10 mg of tissue was homogenized in 1X RIPA buffer (Cell Signaling Technology). Lysates were then sonicated, and the supernatant was removed after centrifugation at 4°C. Protein quantification was performed using Pierce BCA Protein Assay Kit (Thermo Fisher Scientific) as per the manufacturer's protocol. Protein (12.5–40 μg) was loaded and separated by SDS-PAGE. Membranes were then transferred to a polyvinylidene difluoride membrane, blocked in 5% milk solution, and incubated with primary antibodies (listed in Supplementary Table S1) overnight at 4°C. Secondary conjugation was performed using HRP-conjugated anti-rabbit or anti-mouse antibodies (Jackson ImmunoResearch Laboratories), followed by development with chemiluminescent substrate (Thermo Fisher Scientific) and imaged using ChemiDoc Imaging System (Bio-Rad). Uncropped images of blots are shown in Supplementary Figs. S1 and S2.

### Cytokine Array Analysis

Lysates prepared from pancreatic tissue homogenate or conditioned media collected from tumor cells were used for BCA-based protein estimation. Pooled *in vivo* tissue lysates from each group or conditioned media harvested containing an equal amount of protein (300 μg) were subjected to cytokine array profiling as per manufacturer's protocol (ARY006, R&D Systems).

### Flow Cytometry

Pancreatic tumors harvested from PKT mice were enzymatically digested using the solution of 0.6 mg/mL of collagenase P (Roche), 0.8 mg/mL Collagenase V (Sigma-Aldrich), 0.6 mg/mL soybean trypsin inhibitor (Sigma-Aldrich), and 1,800 U/mL DNase I (Thermo Fisher Scientific) in RPMI medium for 20–30 minutes at 37°C. Samples were then washed and resuspended in cold PBS (supplemented with 2 mmol/L Etheylenediaminetetraacetic acid (EDTA) and 0.5% BSA), followed by straining through a 40 μm mesh filter to obtain single-cell suspension. Samples were frozen at −80°C until further use. Prior to flow cytometry staining, samples were thawed, washed, and incubated with mouse FcR blocking reagent (Miltenyi Biotec) before subsequent staining with fluorescently conjugated antibodies listed in Supplementary Table S2. Live/dead cell discrimination was performed using Live/Dead Fixable Blue Dead Cell Stain (Life Technologies), and for intracellular staining, cells were fixed and permeabilized with the Foxp3/Transcription Factor Staining Buffer Set (eBiosciences) as per the manufacturer's instructions. Flow cytometric data acquisition was performed on Cytek Aurora and analyzed using FlowJo v10 software (BD Life Sciences). Gating strategies are depicted in Supplementary Fig. S3.

### Cell Line Treatment

*LSL-Kras^G12D/+^;LSL-Trp53^R172H/+^;Pdx1^Cre/+^* (KPC) mouse-derived primary PDAC tumor cells (6694c2) were generously provided to us by Dr. Ben Z. Stanger (University of Pennsylvania, Philadelphia, PA). As described previously (21, 22), these KPC cells were examined by using the Infectious Microbe PCR amplification test and authenticated. Cells with relatively low passage number (<20) and routinely tested for *Mycoplasma* (InvivoGen Plasmo Test, catalog no. rep-mys-50). For drug treatment studies, KPC PDAC tumor cells were treated in 2.5% FBS RPMI with different concentrations of Uro A (HY-100599; MedChemExpress) or PI3Ki (Omipalisib, HY-10297; MedChemExpress) at indicated timepoint as specified in figure legends. Cell lysates or conditioned media harvested from these cells were used for downstream analysis.

### RNA Isolation and PCR Array Analysis

RNA was isolated from *in vitro* KPC tumor cells using the RNeasy Kit (Qiagen) according to the manufacturer's protocol and as described previously (18). cDNA generated after performing reverse transcription of RNA product was subjected to quantitative PCR analysis using Mouse cancer inflammation and immunity cross-talk array analysis (Qiagen; catalog no.330231).

### T-cell Depletion Experiments

Starting at 4.5 weeks of age, tumor-bearing PKT mice (4.5 weeks old) were treated intraperitoneally with 200 mg/mouse each of anti-CD4^+^ and anti-CD8^+^ T cell–depleting antibodies (BioXCell, anti-CD4 Ab Clone GK1.5 and anti-CD8 Ab Clone 2.43) as described previously. These mice were treated with twice weekly dose for 1 week, and once weekly thereafter with Uro A (20 mg/kg/mouse by oral gavage daily for 5 days per week) or equal volume vehicle control (10% glucose in water) administered by oral gavage until mice were moribund.

### Statistical Analysis

Statistical analysis was performed using Prism Software (GraphPad Software Inc.). Results are shown as values of mean ± SD except where otherwise indicated. Two-tailed Student *t* test was used for the two-group comparison and one-way ANOVA for multiple group comparisons. Determination of significance was made using α cutoff of 0.05. Pairwise comparison of survival curves between individual treatments was performed using the log-rank (Mantel–Cox) test.

### Ethics Approval

All experiments were performed in compliance with the regulations and ethical guidelines for experimental and animal studies of the Institutional Animal Care and Use Committee at the University of Miami (Miami, FL; #15-057 and #18-081).

### Availability of Data and Materials

The data generated in this study are available within the article and its Supplementary Data.

## Results

### Uro A Treatment Downregulates PI3K/AKT/mTOR Signaling, Reduces Primary Tumor Burden, and Alters The Stromal TME in PDAC

**FIGURE 1:**
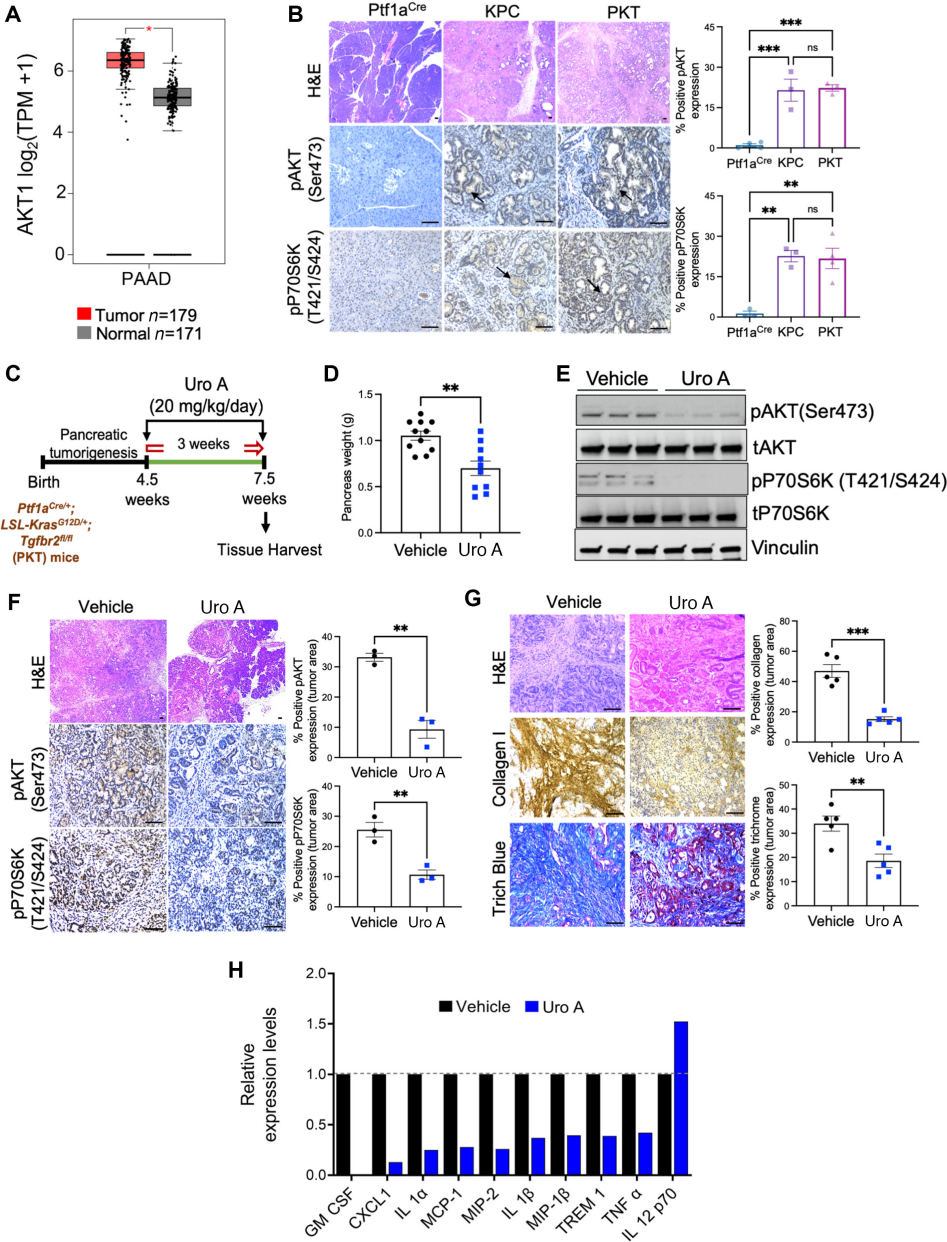
Uro A treatment downregulates PI3K/AKT/mTOR pathway, reduces primary tumor burden, alters the stromal composition and immunomodulatory profile of the TME in a spontaneous murine model of PDAC. **A,** mRNA levels of AKT1 in normal pancreatic tissue (Normal; *n* = 171) and patients with primary PDAC (Tumor; *n* = 179) from GTEx and pancreatic adenocarcinoma (PAAD) TCGA database. **B,** Representative H&E-stained pancreas sections and IHC imaging analysis demonstrating pAKT and pP70S6K expression in the pancreatic tissue sections of non–tumor-bearing Ptf1a^Cre^, and tumor-bearing KPC and PKT GEMMs of PDAC. Quantification of relative areas of positive staining in (within the tumor area) tissue sections from vehicle, and Uro A-treated tumors are indicated in adjacent plots. **C,** Schematic of vehicle or Uro A (20 mg/kg/day) treatment in PKT mice. Treatment was initiated at 4.5 weeks of age when mice reproducibly developed palpable tumors and was continued for 3 weeks before sacrifice. **D,** Pancreas weight analysis of Uro A or vehicle-treated PKT mice. **E,** Western blot analysis showing decreased pAKT and pP70S6K in PKT mice pancreata after Uro A treatment as compared with vehicle-treated PKT mice, *n* = 3 mice/group. **F,** Representative H&E-stained tumor sections, and IHC imaging analysis demonstrating reduction in pAKT and pP70S6K expression within the ductal compartment of pancreatic tissue sections following Uro A treatment in PKT GEMM, *n* = 3 mice/group. **G,** Collagen 1, trichrome blue staining, and their corresponding quantification analysis in pancreatic tumor tissues harvested from PKT mice treated with Uro A or vehicle, *n* = 5 mice/group. **H,** Cytokine array profiling of pancreatic tumor tissues harvested from PKT mice treated with Uro A or vehicle. Scale bar = 50 μm. Individual datapoints with mean ± SEM are shown and compared by two-tailed unpaired *t* test for two group comparison and one-way ANOVA for multiple comparisons; *, *P* < 0.05; **, *P* < 0.01; ***, *P* < 0.001; ^ns^, *P* > 0.05.

Our previous data demonstrate Uro A is effective in PDAC in part through modulation of the PI3K/AKT signaling pathway that is involved in the maintenance of multiple critical features in PDAC tumorigenesis (10). Indeed, our analysis of the publicly available human PDAC The Cancer Genome Atlas (TCGA) and GTEx dataset revealed overexpression of *AKT1* mRNA, a key downstream effector of PI3K, in PDAC samples compared with normal pancreas tissues in healthy individuals (Fig. 1A). In addition, we corroborated these findings using pancreatic tumor sections harvested from GEMMs of PDAC including *LSL-Kras^G12D/+^;LSL-Trp53^R172H/+^;Pdx1^Cre/+^* (KPC) and PKT mice. IHC-based analysis revealed significant hyperactivation of phosphorylated levels of AKT (pAKT; Ser473) and P70S6K (pP70S6K; T421/S424) within the pancreatic tumor ductal compartment of both KPC and PKT as compared with pancreata of non–tumor-bearing Ptf1a^Cre/+^ control mice (Fig. 1B). These results confirmed the involvement of ductal-specific activation of the PI3K/AKT and mTOR signaling axis in PDAC.

Our previous work has established inhibition of PI3K/AKT and mTOR activation in PDAC by Uro A reduces tumor growth and significantly improves overall survival in the PKT GEMM of PDAC. To further delineate stromal immunomodulatory changes with Uro A, PKT mice (*n* = 10–12/arm) were treated with vehicle or Uro A (20 mg/kg/5x per week), and pancreatic tumors were processed for downstream analysis (Fig. 1C). As anticipated, Uro A treatment significantly reduced primary tumor burden in PKT mice compared with the vehicle-treated control group (Fig. 1D; Supplementary Fig. S4A). Importantly, there were no signs of toxic side effects associated with Uro A therapy, as evidenced by the absence of differences in body weight between the vehicle and Uro A-treated groups (Supplementary Fig. S4B). Furthermore, evaluation of tumor tissue lysates by Western blot analysis showed a drastic decrease in the expression levels of phosphorylated (p)AKT (Ser473) and phosphorylated (p)P70S6K (T421/S424) in mice treated with Uro A (Fig. 1E). These findings were corroborated by IHC image analysis, which demonstrated a significant decrease in pAKT and pP70S6K expression within the pancreatic tumor ductal compartment of PKT mice treated with Uro A (Fig. 1F).

The dense desmoplastic stroma is a key characteristic of PDAC tumors, which plays a critical role in conferring resistance to therapy and creating an immunosuppressive environment within the TME (23). To assess the impact of Uro A treatment on PDAC stromal modulation within the TME of PDAC, we used common markers of tissue fibrosis to stain PKT tumor sections from both treatment groups. Our findings indicate that Uro A treatment resulted in a significant decrease in levels of intratumoral collagen I and trichrome staining compared with the vehicle treatment (Fig. 1G). Given the profound antifibrotic changes with Uro A, we next investigated the effect of Uro A on altering the immunomodulatory profile of secreted cytokines within the TME, as these soluble growth factors are known to have a profound influence on stromal activation and the immune composition of PDAC tumors. We performed cytokine array profiling in pancreatic tumor lysates obtained from vehicle and Uro A-treated PKT mice which demonstrated a significant reduction in proinflammatory/immunosuppressive factors including GMCSF, CXCL-1, IL1α, IL1β, and TNFα along with increased expression of IL12, (immunomodulatory cytokine responsible for promoting type 1 immunity) with Uro A treatment (Fig. 1H; Supplementary Fig. S4C). Taken together, our results demonstrate that Uro A treatment is a well-tolerated therapy that effectively reduces primary tumor growth, inhibits PI3K/AKT/mTOR signaling, suppresses intratumoral fibrosis and curtails inflammatory cytokine production in an aggressive mouse model of PDAC.

### Treatment with PI3Ki Omipalisib Recapitulates the Antitumor and Immunomodulatory Benefits of Uro A in PDAC

**FIGURE 2:**
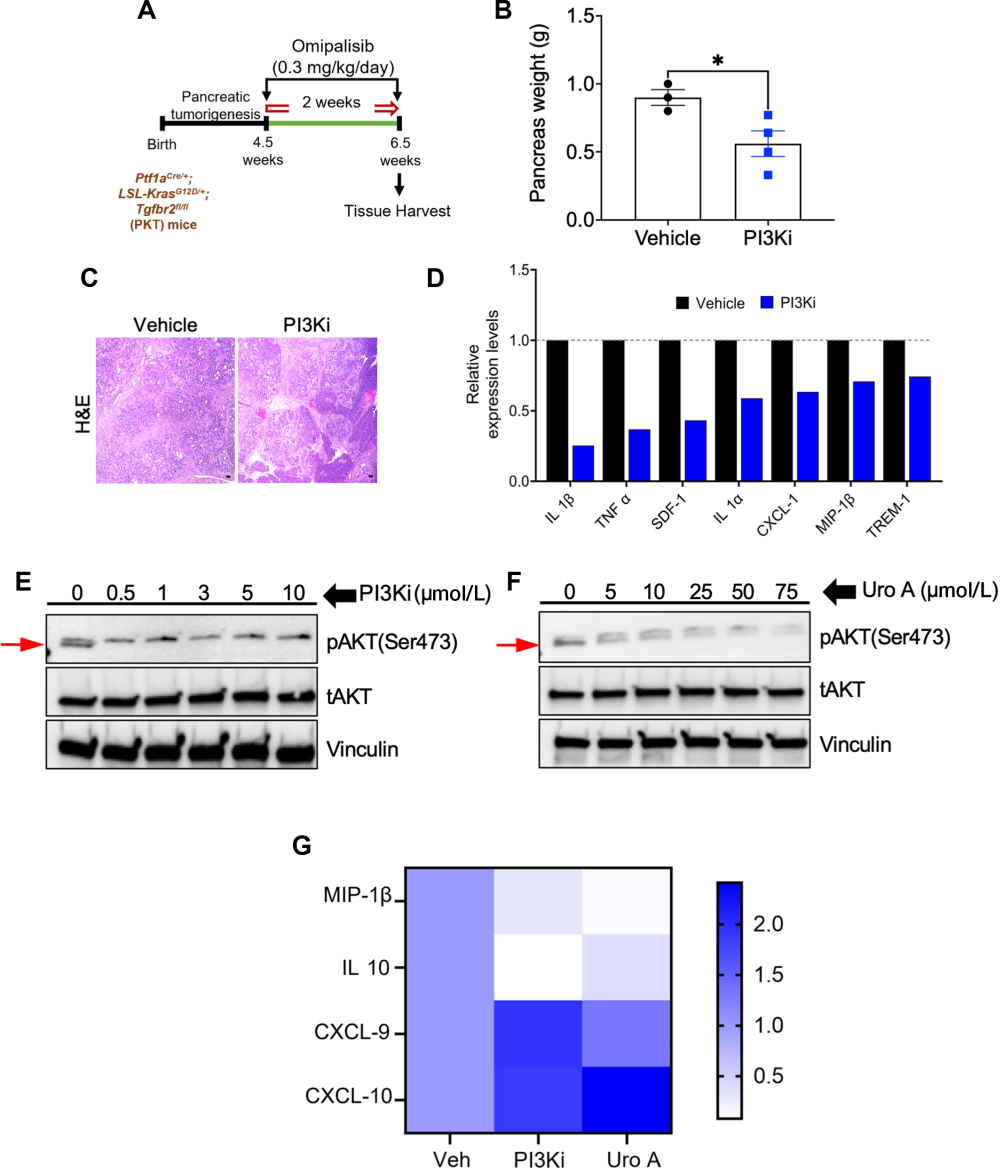
Treatment with PI3Ki (Omipalisib) recapitulates antitumor and immunomodulatory benefits of Uro A in PDAC. **A,** Schematic of PI3Ki (Omipalisib) treatment in PKT mice. **B,** Pancreas weight analysis of PI3Ki- or vehicle-treated PKT mice, *n* = 3–4 mice/group. **C**, Representative H&E-stained tumor sections in PI3Ki-treated mice and vehicle-treated mice. **D,** Cytokine array profiling of pancreatic tumor tissues harvested from PKT mice treated with PI3Ki or vehicle. Western blot analysis demonstrating decreased phosphorylation of AKT (band marked by red arrow) in murine KPC PDAC tumor cell line upon PI3Ki (0–10 μmol/L; **E**) and Uro A (0–75 μmol/L; **F**) treatment for 3 hours. **G,** Representative heat map showing fold change of immunomodulatory gene transcripts (qPCR analysis) associated with PI3Ki (1 μmol/L) or Uro A (25 μmol/L) treatment in KPC PDAC tumor cells for 3 hours. Individual datapoints with mean ± SEM are shown and compared by two-tailed unpaired *t* test; *, *P* < 0.05.

We then aimed to investigate whether pharmacologic inhibition of PI3K/AKT signaling node using clinically validated drug also recapitulates the antitumor and immunomodulatory benefits of Uro A or not, we next examined the effects of Omipalisib (PI3Ki, GSK2126458), a clinically approved potent inhibitor of PI3K/mTOR signaling pathway, on tumor growth and immunomodulation *in vivo*. PKT mice starting at 4.5 weeks of age were treated with vehicle or Omipalisib (PI3Ki, 0.3 mg/kg/daily) for 2 weeks, before sacrifice, and pancreatic tumors were harvested for downstream analysis (Fig. 2A). Like our findings observed with Uro A, Omipalisib-treated PKT mice exhibited a significant reduction in PDAC tumor burden compared with vehicle-treated mice (Fig. 2B). H&E-based histologic assessment of pancreatic tumor sections with PI3Ki revealed a substantial presence of pancreatic acinar cells. In contrast, in the age-matched vehicle-treated PKT mice, the pancreata were completely replaced by 6.5 to 7 weeks (Fig. 2C). In addition, consistent with our intratumoral profile of immunomodulatory cytokines observed with Uro A treatment, PKT tumors treated with PI3Ki significantly attenuated immunosuppressive soluble factors, including IL1β, IL1α, TNFα, CXCL-1, and SDF-1 as compared with vehicle (Fig. 2D; Supplementary Fig. S5A).

We observed similar result *in vitro*, where treatment of KPC cells with increasing doses of either PI3Ki (0.5–10 mmol/L), Uro A (5–75 mmol/L) demonstrated decreased phosphorylation of pAKT with PI3Ki and Uro A, respectively (Fig. 2E and F) and downregulated expression of inflammatory cytokines/chemokines, including (IL10, MCP-5, SDF-1, and CXCL-1) as compared with control (Supplementary Fig. S5B). Furthermore, on comparing the qPCR-based gene expression profile of immunomodulatory genes altered by both Uro A (25 mmol/L)- and PI3Ki (1 mmol/L)-treated cells, our analysis showed downregulation of innate immunosuppressive transcripts, including MIP-1β, IL10 along with increased expression of T-cell chemoattractants including CXCL-9 and CXCL-10 compared with vehicle (Fig. 2G). Overall, these findings suggest that inhibition of PI3K/AKT signaling either using Uro A or pharmacologic inhibitor provides antitumor benefits and alters the immunomodulatory profile of secreted factors in PDAC.

### Uro A Treatment Reduces Immunosuppressive M2-like Macrophages, Facilitates Intratumoral T-cell Infiltration and Controls PDAC Growth in a T Cell–dependent Manner

**FIGURE 3:**
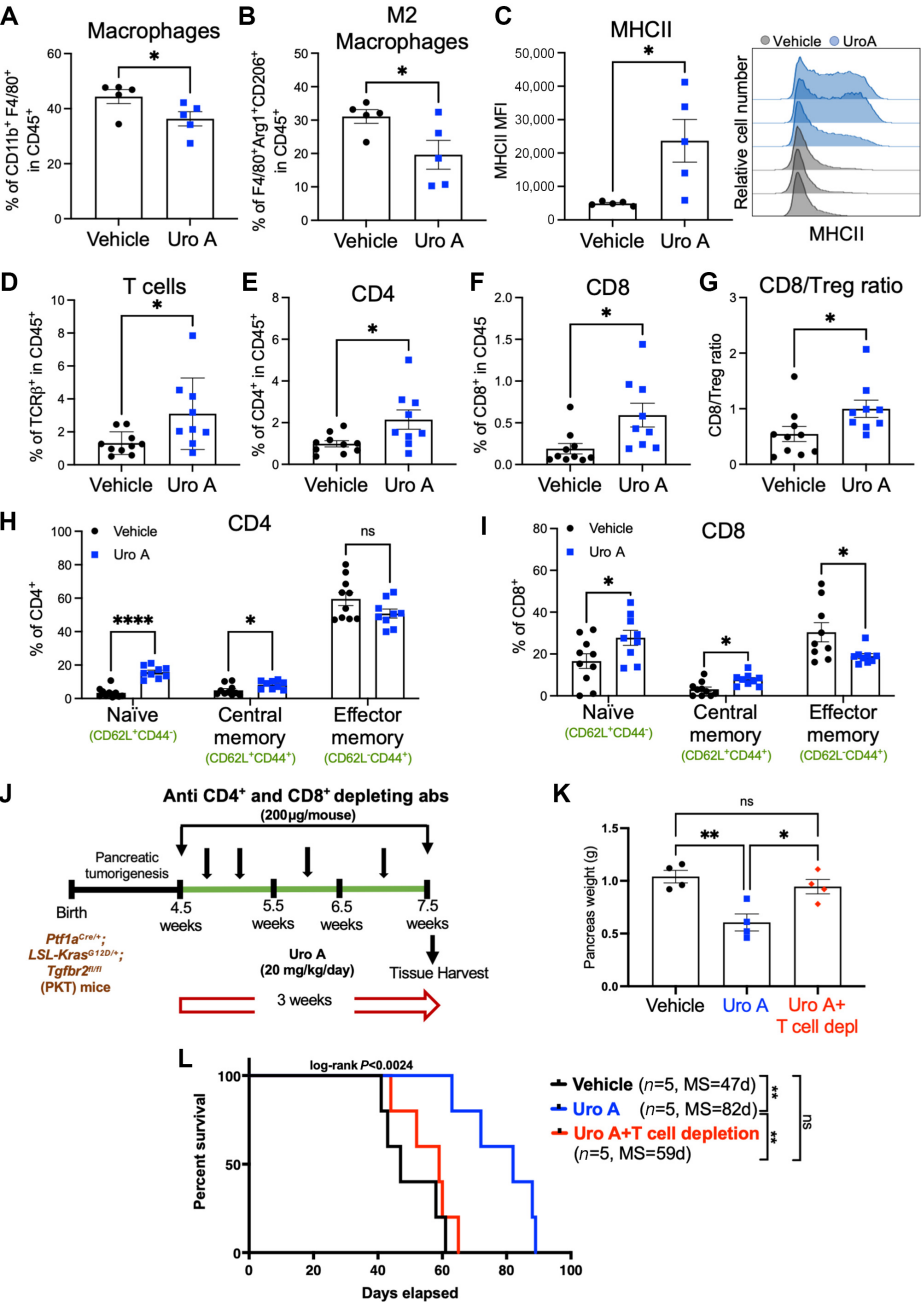
Uro A treatment reduces immunosuppressive TAM populations, facilitates intratumoral T-cell infiltration and controls PDAC growth in a T cell–dependent manner. Single-cell suspensions of pancreatic tumors obtained from PKT mice treated with vehicle or Uro A were subjected to immunophenotyping analysis. Flow cytometric analysis demonstrating significant reduction in the percentage (%) of TAMs (CD11b^+^ F4/80^+^; **A**), and M2-like (F4/80^+^Arg1^+^CD206^+^; **B**) macrophages with Uro A as compared with vehicle mice. **C,** Comparison of mean fluorescence intensity (MFI) of MHCII expression on CD11b^+^ F4/80^+^ macrophages between vehicle- and Uro A-treated mice with representative MHCII histogram (right). Flow cytometric assessment of the tumor-infiltrating adaptive immune compartment depicting frequency of total T cells (CD45^+^ TCRβ^+^; **D**), CD4^+^ T cells (CD45^+^ TCRβ^+^ CD4^+^; **E**), CD8^+^ T cells (CD45^+^ TCRβ^+^ CD8^+^; **F**), CD8/T_reg_ ratio (**G**), and frequency of naïve (CD62L^+^ CD44^−^), central memory (CD62L^+^ CD44^+^), and effector memory (CD62L^−^ CD44^+^) in CD4^+^ (**H**) and CD8^+^ (**I**) T cells in mice treated with vehicle or Uro A. **J,** Schematic representation of PKT GEMM treated with vehicle, Uro A, or Uro A following T-cell depletion with anti-CD4 and anti-CD8 antibodies. **K,** Pancreas weight comparison among vehicle- and Uro A-treated mice with or without T-cell depletion. **L,** Kaplan–Meier plot and log-rank test analysis showing impaired antitumor efficacy of Uro A treatment in PKT mice subjected to T-cell depletion compared with non–T cell–depleted Uro A- and vehicle-treated mice. Individual datapoints with mean ± SEM are shown and compared by two-tailed unpaired *t* test. *, *P* < 0.05; **, *P* < 0.01; ****, *P* < 0.0001; ^ns^, *P* > 0.05.

Next, we investigated the effects of Uro A treatment on the immune microenvironment of PDAC tumors *in vivo.* We conducted flow cytometric analysis of PKT tumors and found a decrease in the frequency of total macrophages (Fig. 3A), along with a significant reduction in the immunosuppressive M2-like macrophage population (Fig. 3B) after treatment with Uro A. Interestingly, macrophages from Uro A-treated tumors demonstrated an increased expression of the antigen-presenting molecule, MHC class II, when compared with macrophages from vehicle-treated tumors (Fig. 3C). This innate immune remodeling within the macrophage subsets after Uro A treatment was associated with a significant increase in the frequency of total T cells (Fig. 3D), including both CD4^+^ (Fig. 3E) and CD8^+^ (Fig. 3F) T-cell populations. In addition, we observed a significant increase in the CD8/regulatory T cell (Treg) ratio in tumors from PKT mice treated with Uro A (Fig. 3G). Intriguingly, upon further investigation of the T-cell populations by flow cytometric analysis, we found a significant increase in the proportion of naïve (CD62L^+^CD44^−^) and central memory (CD62L^+^CD44^+^) cells within the CD4^+^ (Fig. 3H) and CD8^+^ (Fig. 3I) T-cell compartments, along with a modest decrease in the effector memory CD8^+^ T cells in the tumors of Uro A-treated mice compared with vehicle-treated controls. Notably, memory T cells, including central memory T cells, have been previously established to possess superior persistence and antitumor immunity compared with effector memory T cells (24). Therefore, the profound antitumor benefits observed with Uro A could be associated with the expansion of these immune subsets within the TME of PDAC.

After observing significant immunologic changes within the T-cell compartment, we investigated whether the observed antitumor effects of Uro A treatment were T-cell dependent. To test this, we depleted T cells using anti-CD4 and anti-CD8 antibodies (200 mg/mouse) in 4.5 weeks old PKT mice treated with vehicle or Uro A, and pancreatic tumors were harvested for endpoint analysis (after 3 weeks), or the treatment was continued until mice were moribund (Fig. 3J). The PKT mice with intact T-cell populations treated with Uro A displayed a significant reduction in tumor weight compared with those treated with vehicle. In contrast, T cell–depleted mice treated with Uro A did not show any significant reduction in tumor weight compared with vehicle, demonstrating the effects of Uro A are dependent on T-cell function (Fig. 3K). Furthermore, we also observed that T-cell depletion in Uro A-treated mice resulted in a drastic reduction in overall survival with higher tumor burden as compared with Uro A-treated PKT mice with intact T-cell populations (*P* = 0.0024, log-rank; Fig. 3L). These results suggest that Uro A treatment increases the frequencies of CD4^+^ and CD8^+^ T cells with memory-like phenotype within the TME and that the associated antitumor benefits of Uro A treatment are T-cell dependent in PKT mice.

### Uro A Treatment has Opposite Effect on PD-1 and PD-L1 Expression within Intratumoral T Cells and Macrophages

**FIGURE 4:**
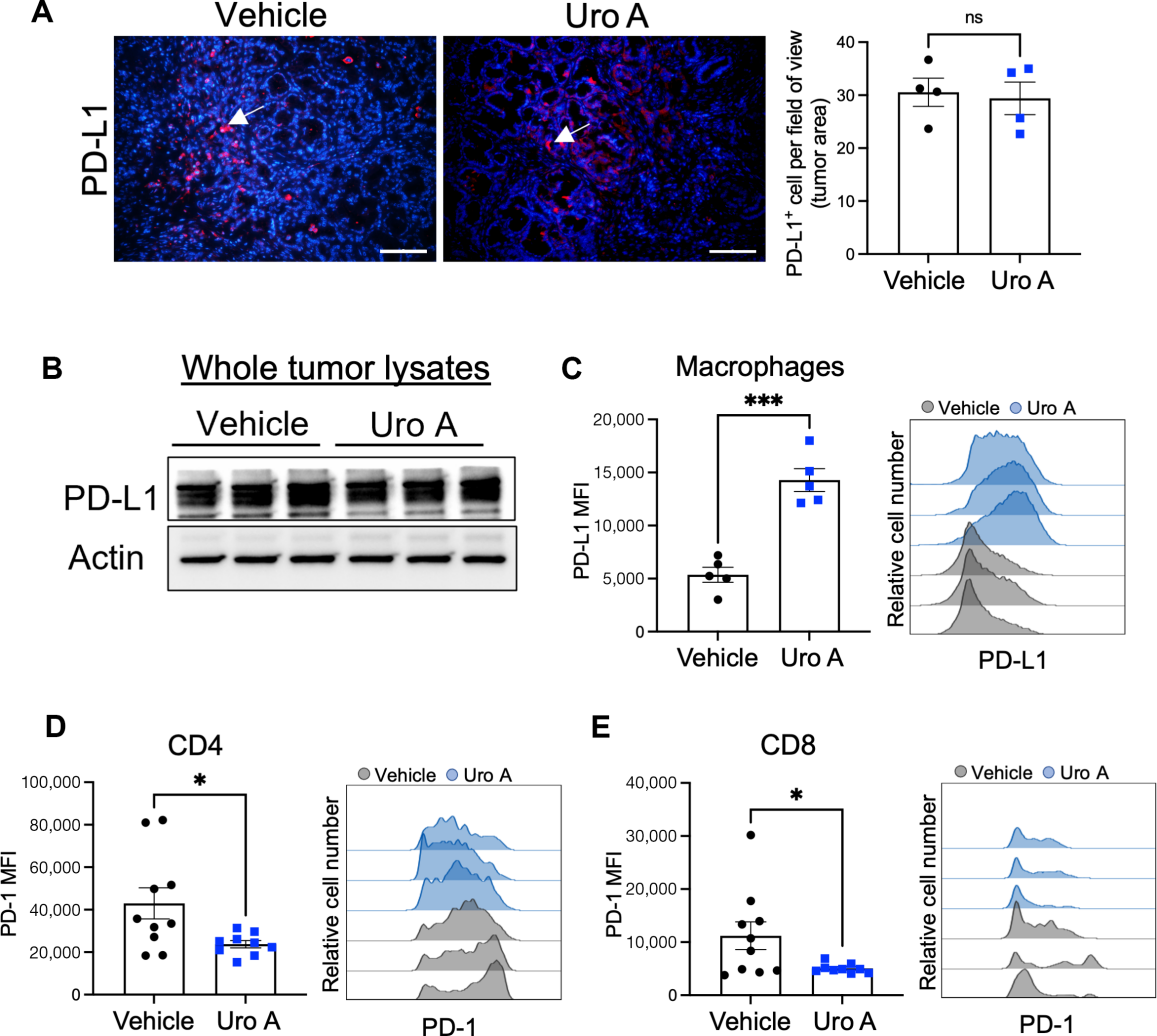
PD-1/PD-L1 expression in the PDAC TME with Uro A treatment. **A,** Immunofluorescence-based histologic analysis depicting PD-L1–positive cells within pancreatic tumor sections harvested from vehicle or Uro A-treated PKT mice. Mean of positive cells per field of view within the tumor area for each mouse is shown in adjacent plot, *n* = 4 mice per group. **B,** Western blot analysis demonstrating PD-L1 protein expression in whole pancreatic tumor lysates from vehicle- or Uro A-treated PKT mice. **C,** Flow cytometric analysis of PD-L1 expression in the macrophage population with representative PD-L1 histogram overlay of vehicle- and Uro A-treated PKT mice (right). Flow cytometric analysis of PD-1 expression on CD4^+^ T cells (**D**) and CD8^+^ T cells (**E**) with representative PD-1 histogram overlay of vehicle- and Uro A-treated groups (right) of pancreatic tumors from PKT mice. Individual datapoints with mean ± SEM are shown and compared by two-tailed unpaired *t* test. *, *P* < 0.05; ***, *P* < 0.001; ^ns^, *P* > 0.05; MFI, mean fluorescence intensity.

The efficacy of immunotherapy depends significantly on the infiltration of cytotoxic T cells and the presence of the PD-1/PD-L1 signaling axis within the TME (25, 26). Therefore, we next aimed to determine changes in the expression of the PD1/PD-L1 axis within the TME produced by Uro A treatment to assess the potential efficacy of combinatorial PD-1 blockade with Uro A treatment. Immunofluorescence staining of PKT tumor sections (Fig. 4A), and Western blot analysis of whole tumor lysates (Fig. 4B) showed no difference in PD-L1 expression, between vehicle and Uro A-treated groups. Similar results were also obtained when we compared PD-L1 expression on KPC PDAC cells (*in vitro*) treated with Uro A or PI3Ki (Supplementary Fig. S6A and S6B). In contrast, using flow cytometry analysis, we observed increased expression of PD-L1 on intratumoral macrophages from Uro A-treated PKT mice (Fig. 4C). Interestingly, evaluation of the T-cell compartment by flow cytometry revealed a significant decrease in PD-1 expression within both CD4^+^ (Fig. 4D) and CD8^+^ (Fig. 4E) T cells, consistent with the increase in naïve/memory phenotype we observed following Uro A treatment (Fig. 3). Despite the reduction in PD-1 MFI on T cells, we still observed a substantial proportion of T cells (mostly CD4^+^ cells) expressed this receptor within the PDAC TME (Fig. 4D and E, right), thereby providing a strong rationale to evaluate the therapeutic synergy of Uro A in combination with aPD-1 immunotherapy.

### Uro A Treatment Combined with aPD-1 Blockade Significantly Improves Overall Survival in PKT Mice and is Associated with Increased Intratumoral Infiltration of Effector CD4^+^ Th 1 Cells

**FIGURE 5:**
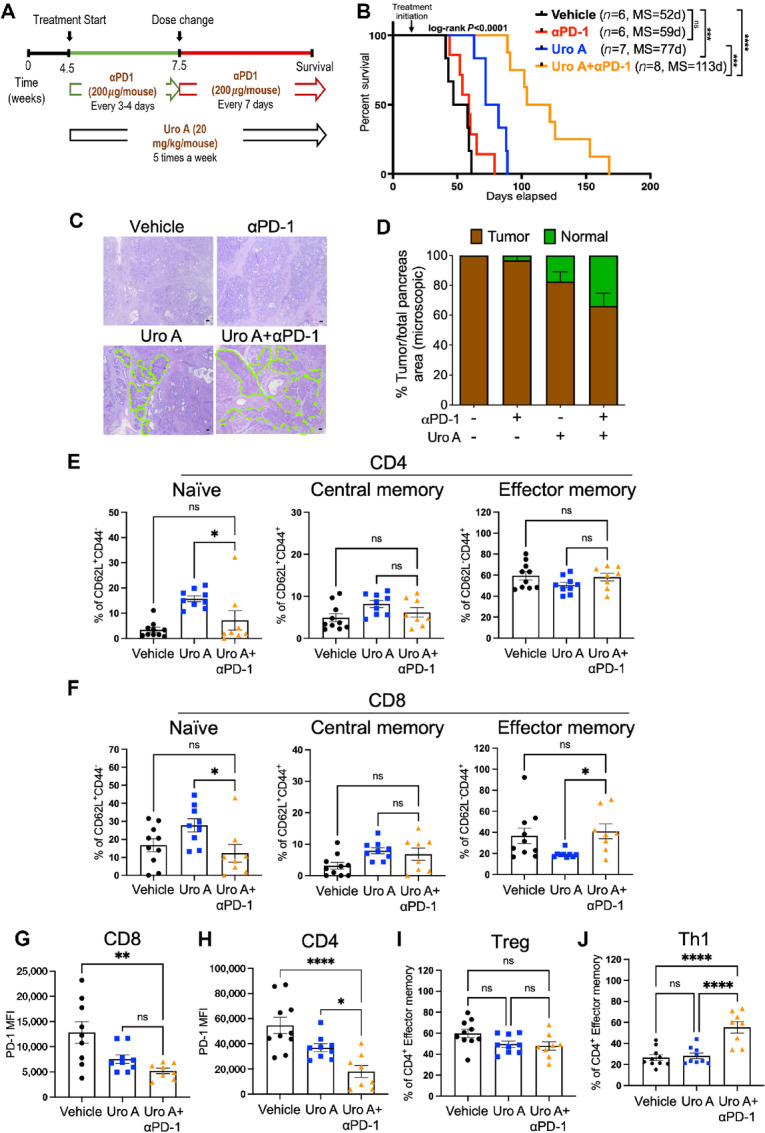
Addition of αPD-1 blockade to Uro A treatment further decreases PDAC tumor burden and improves overall survival in PKT mice. **A,** Schematic representation of PKT mice treated with vehicle, Uro A, or Uro A in combination with aPD-1. **B,** Kaplan–Meier plot and log-rank test analysis showing superior survival of PKT mice treated with Uro A in combination with aPD-1 when compared with all other groups. **C,** Representative H&E-stained tumor sections after 3 weeks of drug treatment depicting significant presence of normal pancreatic architecture (highlighted in green) in PKT mice treated with combination therapy of Uro A and αPD-1 as compared with other treatment cohorts (scale bar = 50 μm). **D,** Percentage of tumor area (brown bar) relative to the normal pancreas (green bar) quantified within the whole pancreas, by capturing multiple fields of view in lower magnification. **E–J,** Flow cytometric assessment of the tumor-infiltrating adaptive immune compartment in mice treated with vehicle, Uro A (same mice cohort used in previous figures) or Uro A+aPD-1 depicting frequency of CD4^+^ (**E**) and CD8^+^ (**F**) T cells naïve, central memory, and effector memory populations. PD-1 MFI expression in CD8^+^ T cells (**G**) and PD-1 MFI expression in CD4^+^ T cells (**H**). Flow cytometric analysis of tumor-infiltrating effector memory CD4^+^ T cells showing frequency of Tregs (**I**), and Th1 (**J**) cells in mice treated with vehicle, Uro A or Uro A+aPD-1. Individual datapoints with mean ± SEM are shown and compared by one-way ANOVA test. *, *P* < 0.05; **, *P* < 0.01; ***, *P* < 0.001; ****, *P* < 0.0001; ^ns^, *P* > 0.05.

On the basis of the favorable immunologic changes seen in both the innate and adaptive immune subsets with Uro A treatment, we then sought to investigate whether combining Uro A with PD-1 blockade therapy would lead to synergistic improvement in overall survival. To test this hypothesis, PKT GEMM (*n* = 6–8/arm) were treated with either vehicle, aPD-1 antibody (200 μg/mouse), Uro A (20 mg/kg/daily), or Uro A in combination with anti-PD1 antibody (Uro A+aPD-1) until moribund (Fig. 5A).

Consistent with the lack of response to aPD-1 monotherapy in PDAC (19), we observed minimal survival benefit with aPD1 antibody alone compared with vehicle treatment [*P* = 0.206, log-rank, median survival (MS) = 59 vs. 52 days]. The addition of PD-1 blockade to Uro A treatment exhibited the most profound improvement in overall survival compared with aPD-1 monotherapy (*P* < 0.0001, MS = 113 vs. 59 days), or Uro A alone (*P* = 0.0002, log-rank, MS = 113 vs. 77 days; Fig. 5B). Importantly, PKT mice exhibited minimal body weight reduction with Uro A+aPD-1 therapy when compared with Uro A alone (Supplementary Fig. S7), indicating that combined treatment is well tolerated with minimal toxicity in this preclinical model of PDAC. Analysis of the histologic architecture in H&E-stained pancreatic tumor sections revealed a near 100% tumor penetrance in vehicle-treated PKT mice or aPD-1 monotherapy group with substantial presence of late PanINs, tumor cells, and dense stromal architecture. Notably, Uro A treatment alone or in combination with aPD-1 therapy, depicted a higher presence of normal acinar cells (highlighted in green), Acinar to ductal metaplasia (ADMs), and early PanIN lesions, compared with other treatment cohorts (Fig. 5C and D).

Given the remarkable role of Uro A in providing protective antitumor immunity and its profound survival benefits in combination with aPD-1 immunotherapy, we aimed to investigate whether Uro A+aPD-1 treatment leads to any additional changes in the phenotype of the adaptive immune CD4^+^ and CD8^+^ T-cell compartments. We performed flow cytometric analysis on PKT tumors treated for 3 weeks with Uro A+aPD-1 and observed a significant reduction in naïve (CD62L^+^ CD44^−^) CD4^+^ and CD8^+^ T cells along with a concomitant increase in effector memory (CD62L^−^ CD44^+^) CD8^+^ T cells when compared with Uro A monotherapy (Fig. 5E and F). On the basis of these results, we further examined CD8^+^, as well as CD4^+^ effector memory cell subsets and noted reduced expression of PD-1 on these immune cells with the Uro A+aPD-1 therapy as compared with vehicle or Uro A treatment alone (Fig. 5G and H).

Upon T-cell receptor activation, naïve CD4 T cells can differentiate into distinct Th lineages with protumoral or antitumoral activity and actively shape tumor immunity (27). In PKT mice, flow cytometric analysis of tumor-infiltrating effector memory CD4^+^ T cells using lineage-specific markers revealed no significant difference in the frequency of immunosuppressive Treg (CD4^+^CD62L^−^CD44^+^Foxp3^high^) population among the treatment cohorts (Fig. 5I). However, we observed a significant increase in the frequency of antitumor effector memory Th1 cells (CD4^+^CD62L^−^CD44^+^Tbet^+^) within the TME of PKT mice treated with Uro A+aPD-1 therapy compared with vehicle or Uro A treatment alone (Fig. 5J). Taken together, these results demonstrate that combining Uro A with aPD1 improves survival and promotes the expansion of CD4^+^ Th1 immune subsets within the TME of PDAC.

## Discussion

**FIGURE 6:**
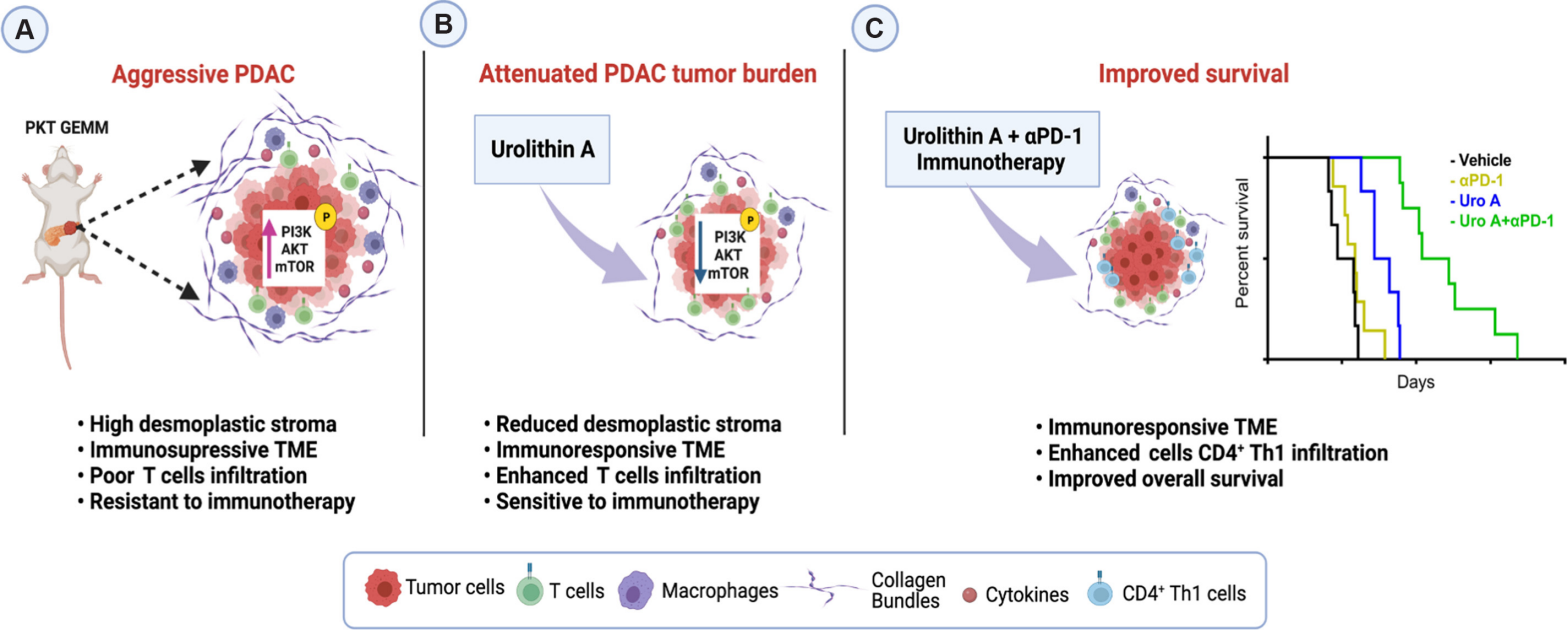
Uro A remodels stromal immune microenvironment and improves overall survival with aPD-1 therapy. **A,** Presence of dense desmoplastic stroma, hyperactivation of PI3K/AKT/mTOR signaling, and T cell excluded immunosuppressive TME in an aggressive PKT GEMM of PDAC. **B,** Treatment with Uro A led to significant attenuation of primary tumor burden, inhibition of PI3K/AKT/mTOR pathway, reduction of stromal fibrotic composition, and enhanced T-cell infiltration. **C,** Uro A sensitizes PKT PDAC tumors to aPD-1 immunotherapy, promoting the expansion of effector CD4^+^ Th1 subsets and substantially improving survival outcomes. [Image created with BioRender license agreement number JU25GQ83NM]

Pancreatic cancer has remained a major therapeutic challenge due to their profound stromal desmoplasia and uniquely immunosuppressive TME (23, 28). Previously, we have shown that Uro A, a natural gut microbial metabolite which is also involved in enhancement of gut barrier integrity can downregulate the oncogenic PI3K/AKT/mTOR signaling pathway, induce cell-cycle arrest, and increase apoptosis in cancer cells (10). In the current study, we demonstrate that Uro A suppresses intratumoral fibrosis and attenuates immunosuppression by impacting the infiltration of TAMs and invigorating a T cell–dependent antitumor response in PKT GEMM of PDAC. Furthermore, our results support Uro A in combination with ICB as a potential treatment strategy in PDAC, where therapeutic options remain limited (Fig. 6). Several dietary and naturally occurring polyphenols have been tested as adjuvant therapies due to their potent antioxidant and anti-inflammatory properties that enhance gut barrier functions (29–31). Numerous studies have established the protective effects of Uro A in suppressing fibroinflammatory responses by influencing different cellular signaling pathways (32, 33). Our data show that Uro A effectively targets the PI3K/AKT/mTOR signaling pathway leading to a significant reduction in intratumoral fibrosis and a decrease in proinflammatory/immunosuppressive cytokines in the TME.

Immunosuppressive TAMs promote T-cell exclusion within the TME, fostering a major resistance mechanism to the effectiveness of immunotherapy in PDAC (23). TAMs skewed toward an M2-like phenotype not only secrete cytokines such as IL10 and TGFβ that suppresses the activity of intratumoral T cells, but also promote the formation of a dense extracellular matrix around the tumor, which act as a physical barrier preventing effector T cells and therapeutic compounds from penetrating the tumor (34). Our data in PKT tumors show that Uro A treatment is associated with a reduced population of M2-like macrophages and a concurrent increase in the infiltration of CD4^+^ and CD8^+^ T cells. Notably, the antitumor benefits of Uro A were completely abolished when T-cell populations were depleted using anti-CD4 and anti-CD8 antibodies, indicating the crucial role of T cells in coordinating the antitumor functions of Uro A. Several studies have demonstrated that both CD4^+^ and CD8^+^ T cells can promote antitumor immunity by directly killing tumor cells or indirectly activating innate immune cells to mount a robust antitumor response (35). However, the precise role of Uro A on specific T-cell subsets and their interaction with the other diverse immune constituents of the PDAC TME are mainly unknown and merit further investigation.

Phenotypic characterization of intratumoral CD4 and CD8 T cells using the classical markers, CD44 and CD62L, demonstrated that Uro A treatment promotes the infiltration of T cells with naïve and central memory phenotype. Studies have shown that memory T cells, including central memory T cells, possess superior antitumor efficacy and long-lasting persistence compared with effector T cells (24). Notably, a recent study by Denk and colleagues, has reported a direct impact of Uro A on the expansion of CD8^+^CD44^low^CD62L^high^Sca1^high^ T stem cell memory phenotype with enhanced antitumor function in colon cancer (36). Contrary to our findings, Denk and colleagues reported no significant changes in the CD4^+^ T-cell compartment after Uro A treatment, indicating that the immunomodulatory effects of Uro A could vary among different types of solid tumors. Also noteworthy is that stem cell memory T cells represent a subset of minimally differentiated T cells, which share the phenotype CD44^low/neg^ CD62L^high^ with naïve and central memory T cells, therefore, in-depth functional analysis to further delineating the role of each of these adaptive immune subsets with Uro A would be worthy to pursue in PDAC.

Recent developments in the field have highlighted that the response to ICB is a complex process, which can be characterized by the heterogeneity of CD8^+^ T cells, based on their expression levels of PD-1. Studies have now established that CD8^+^ T cells exhibiting memory-like features, and expressing low levels of PD-1, are the most effective responders to ICB, leading to the development of terminally differentiated cells (37). Upon evaluation of the exhaustion marker PD-1 in intratumoral cells of PKT mice, Uro A treatment resulted in a significant reduction of PD-1 expression in both CD8^+^ and CD4^+^ when compared with vehicle controls. This characteristic may in part explain why we observed such a profound effect on the survival of PKT mice treated with aPD-1 antibody with Uro A.

Several studies have highlighted the significance of PD-L1 expression on antigen-presenting cells in regulating the immune response (38, 39). Recently, Liu and colleagues has established a correlation between elevated levels of PD-L1 expression on macrophages and improved overall survival rates among a cohort of patients with lung cancer undergoing PD-1 therapy (40). Notably, our analysis of tumors treated with Uro A showed significantly higher levels of PD-L1 expression on macrophages compared with those treated with the vehicle. This finding suggests an additional explanation for the observed synergy between Uro A and aPD-1 therapy in the study.

In addition, a previous study showed that combination therapy with ICB improves the survival of murine PDAC models by enhancing Th1- and CD8^+^-mediated immune responses (41). Consistent with these findings, our results show that the addition of aPD-1 to Uro A treatment significantly improves survival in a highly aggressive GEMM of PDAC and is accompanied by the expansion of CD4^+^ Th1 and CD8^+^ effector T cells with reduced expression of PD-1. The cellular mechanisms by which Uro A results in this observed phenotype should thus be explored in future studies, as this could uncover additional therapeutic vulnerabilities that could enhance the function of Uro A as an immune adjuvant.

In conclusion, we demonstrate that Uro A effectively reduces primary tumor burden in a T cell–dependent manner, attenuates immunosuppressive M2-like TAMs, and significantly increases the infiltration of CD4^+^ and CD8^+^ T cells with a memory-like phenotype in the PDAC TME. Furthermore, our research highlights that combining Uro A and aPD-1 therapy leads to a robust antitumor response, significantly improves survival and promotes the expansion of effector CD4^+^ Th1 cells in the preclinical mouse model of PDAC. Because immunotherapeutic drugs have had limited success in improving outcomes for patients with PDAC, our work provides valuable preclinical data for the potential clinical translation of Uro A as an adjunct therapy to enhance the sensitivity of ICB in patients with PDAC.

## Supplementary Material

Figure S1 Raw uncropped images of Western blot membranes for Figs.1e and 3d.

Figure S2 Raw uncropped images of Western blot membranes for Figs. 2e, 2f, S3a and S3b.

Figure S3 Gating strategies used for flow cytometry analysis.

Figure S4 Tumor weight, mouse weight and cytokine profiling of Uro A treatment in PKT mice.

Figure S5 Cytokine profiling of PKT tumors and KPC cells treated with PI3Ki or Uro A.

Figure S6 PD-L1 expression in Uro A or PI3Ki treated KPC cells.

Figure S7 Recorded body weights of PKT mice treated with Uro A, aPD-1 and Uro A+aPD-1.

Supplementary Tables S1-S2 Primary antibodies for flow cytometry, immunohistochemistry and Western blot analysis.

## References

[1] Siegel RL , Miller KD, Jemal A. Cancer statistics, 2018. CA Cancer J Clin 2018;68:7–30. 29313949 10.3322/caac.21442

[2] Miller KD , Goding Sauer A, Ortiz AP, Fedewa SA, Pinheiro PS, Tortolero-Luna G, . Cancer statistics for Hispanics/Latinos, 2018. CA Cancer J Clin 2018;68:425–45. 30285281 10.3322/caac.21494

[3] Rahib L , Smith BD, Aizenberg R, Rosenzweig AB, Fleshman JM, Matrisian LM. Projecting cancer incidence and deaths to 2030: the unexpected burden of thyroid, liver, and pancreas cancers in the United States. Cancer Res 2014;74:2913–21. 24840647 10.1158/0008-5472.CAN-14-0155

[4] Tang J , Yu JX, Hubbard-Lucey VM, Neftelinov ST, Hodge JP, Lin Y. Trial watch: the clinical trial landscape for PD1/PDL1 immune checkpoint inhibitors. Nat Rev Drug Discov 2018;17:854–5. 30482962 10.1038/nrd.2018.210

[5] Marabelle A , Le DT, Ascierto PA, Di Giacomo AM, De Jesus-Acosta A, Delord JP, . Efficacy of pembrolizumab in patients with noncolorectal high microsatellite instability/mismatch repair-deficient cancer: results from the phase II KEYNOTE-158 study. J Clin Oncol 2020;38:1–10. 31682550 10.1200/JCO.19.02105 PMC8184060

[6] O'Reilly EM , Oh DY, Dhani N, Renouf DJ, Lee MA, Sun W, . Durvalumab with or without tremelimumab for patients with metastatic pancreatic ductal adenocarcinoma: a phase 2 randomized clinical trial. JAMA Oncol 2019;5:1431–8. 31318392 10.1001/jamaoncol.2019.1588 PMC6647002

[7] Balachandran VP , Beatty GL, Dougan SK. Broadening the impact of immunotherapy to pancreatic cancer: challenges and opportunities. Gastroenterology 2019;156:2056–72. 30660727 10.1053/j.gastro.2018.12.038 PMC6486864

[8] Collins MA , Bednar F, Zhang Y, Brisset JC, Galbán S, Galbán CJ, . Oncogenic Kras is required for both the initiation and maintenance of pancreatic cancer in mice. J Clin Invest 2012;122:639–53. 22232209 10.1172/JCI59227 PMC3266788

[9] di Magliano MP , Logsdon CD. Roles for KRAS in pancreatic tumor development and progression. Gastroenterology 2013;144:1220–9. 23622131 10.1053/j.gastro.2013.01.071 PMC3902845

[10] Totiger TM , Srinivasan S, Jala VR, Lamichhane P, Dosch AR, Gaidarski AA 3rd, . Urolithin A, a novel natural compound to target PI3K/AKT/mTOR pathway in pancreatic cancer. Mol Cancer Ther 2019;18:301–11. 30404927 10.1158/1535-7163.MCT-18-0464 PMC6363854

[11] Thibault B , Ramos-Delgado F, Pons-Tostivint E, Therville N, Cintas C, Arcucci S, . Pancreatic cancer intrinsic PI3Kα activity accelerates metastasis and rewires macrophage component. EMBO Mol Med 2021;13:e13502. 34033220 10.15252/emmm.202013502 PMC8261517

[12] Fruman DA , Rommel C. PI3K and cancer: lessons, challenges and opportunities. Nat Rev Drug Discov 2014;13:140–56. 24481312 10.1038/nrd4204 PMC3994981

[13] Mehra S , Deshpande N, Nagathihalli N. Targeting PI3K pathway in pancreatic ductal adenocarcinoma: rationale and progress. Cancers 2021;13:4434. 34503244 10.3390/cancers13174434 PMC8430624

[14] Mehra S , Srinivasan S, Singh S, Zhou Z, Garrido V, Silva IDC, . Urolithin A attenuates severity of chronic pancreatitis associated with continued alcohol intake by inhibiting PI3K/AKT/mTOR signaling. Am J Physiol Gastrointest Liver Physiol 2022;323:G375–86. 36098401 10.1152/ajpgi.00159.2022 PMC9602784

[15] Nagathihalli NS , Castellanos JA, Lamichhane P, Messaggio F, Shi C, Dai X, . Inverse correlation of STAT3 and MEK signaling mediates resistance to RAS pathway inhibition in pancreatic cancer. Cancer Res 2018;78:6235–46. 30154150 10.1158/0008-5472.CAN-18-0634 PMC6878978

[16] Dosch AR , Dai X, Reyzer ML, Mehra S, Srinivasan S, Willobee BA, . Combined Src/EGFR inhibition targets STAT3 signaling and induces stromal remodeling to improve survival in pancreatic cancer. Mol Cancer Res 2020;18:623–31. 31949002 10.1158/1541-7786.MCR-19-0741 PMC7127944

[17] Ijichi H , Chytil A, Gorska AE, Aakre ME, Fujitani Y, Fujitani S, . Aggressive pancreatic ductal adenocarcinoma in mice caused by pancreas-specific blockade of transforming growth factor-beta signaling in cooperation with active Kras expression. Genes Dev 2006;20:3147–60. 17114585 10.1101/gad.1475506 PMC1635149

[18] Dosch AR , Singh S, Dai X, Mehra S, Silva IC, Bianchi A, . Targeting tumor-stromal IL6/STAT3 signaling through IL1 receptor inhibition in pancreatic cancer. Mol Cancer Ther 2021;20:2280–90. 34518296 10.1158/1535-7163.MCT-21-0083 PMC8571047

[19] Datta J , Dai X, Bianchi A, De Castro Silva I, Mehra S, Garrido VT, . Combined MEK and STAT3 inhibition uncovers stromal plasticity by enriching for cancer-associated fibroblasts with mesenchymal stem cell-like features to overcome immunotherapy resistance in pancreatic cancer. Gastroenterology 2022;163:1593–612. 35948109 10.1053/j.gastro.2022.07.076 PMC10257389

[20] Nagathihalli NS , Castellanos JA, Shi C, Beesetty Y, Reyzer ML, Caprioli R, . Signal transducer and activator of transcription 3, mediated remodeling of the tumor microenvironment results in enhanced tumor drug delivery in a mouse model of pancreatic cancer. Gastroenterology 2015;149:1932–43. 26255562 10.1053/j.gastro.2015.07.058 PMC4863449

[21] Datta J , Bianchi A, De Castro Silva I, Deshpande NU, Cao LL, Mehra S, . Distinct mechanisms of innate and adaptive immune regulation underlie poor oncologic outcomes associated with KRAS-TP53 co-alteration in pancreatic cancer. Oncogene 2022;41:3640–54. 35701533 10.1038/s41388-022-02368-w

[22] Li J , Byrne KT, Yan F, Yamazoe T, Chen Z, Baslan T, . Tumor cell-intrinsic factors underlie heterogeneity of immune cell infiltration and response to immunotherapy. Immunity 2018;49:178–93. 29958801 10.1016/j.immuni.2018.06.006 PMC6707727

[23] Ligorio M , Sil S, Malagon-Lopez J, Nieman LT, Misale S, Di Pilato M, . Stromal microenvironment shapes the intratumoral architecture of pancreatic cancer. Cell 2019;178:160–75. 31155233 10.1016/j.cell.2019.05.012 PMC6697165

[24] Klebanoff CA , Gattinoni L, Torabi-Parizi P, Kerstann K, Cardones AR, Finkelstein SE, . Central memory self/tumor-reactive CD8^+^ T cells confer superior antitumor immunity compared with effector memory T cells. Proc Natl Acad Sci U S A 2005;102:9571–6. 15980149 10.1073/pnas.0503726102 PMC1172264

[25] Saka D , Gökalp M, Piyade B, Cevik NC, Arik Sever E, Unutmaz D, . Mechanisms of T-cell exhaustion in pancreatic cancer. Cancers 2020;12:2274. 32823814 10.3390/cancers12082274 PMC7464444

[26] Davis AA , Patel VG. The role of PD-L1 expression as a predictive biomarker: an analysis of all US Food and Drug Administration (FDA) approvals of immune checkpoint inhibitors. J Immunother Cancer 2019;7:278. 31655605 10.1186/s40425-019-0768-9 PMC6815032

[27] Kim H-J , Cantor H. CD4 T-cell subsets and tumor immunity: the helpful and the not-so-helpful. Cancer Immunol Res 2014;2:91–8. 24778273 10.1158/2326-6066.CIR-13-0216

[28] Beatty GL , Eghbali S, Kim R. Deploying immunotherapy in pancreatic cancer: Defining mechanisms of response and resistance. Am Soc Clin Oncol Educ Book 2017;37:267–78. 28561678 10.1200/EDBK_175232

[29] Saha P , Yeoh BS, Singh R, Chandrasekar B, Vemula PK, Haribabu B, . Gut microbiota conversion of dietary ellagic acid into bioactive phytoceutical Urolithin A inhibits heme peroxidases. PLoS One 2016;11:e0156811. 27254317 10.1371/journal.pone.0156811 PMC4890745

[30] Komatsu W , Kishi H, Yagasaki K, Ohhira S. Urolithin A attenuates pro-inflammatory mediator production by suppressing PI3-K/Akt/NF-κB and JNK/AP-1 signaling pathways in lipopolysaccharide-stimulated RAW264 macrophages: possible involvement of NADPH oxidase-derived reactive oxygen species. Eur J Pharmacol 2018;833:411–24. 29932926 10.1016/j.ejphar.2018.06.023

[31] Savi M , Bocchi L, Sala R, Frati C, Lagrasta C, Madeddu D, . Parenchymal and stromal cells contribute to pro-inflammatory myocardial environment at early stages of diabetes: protective role of resveratrol. Nutrients 2016;8:729. 27854328 10.3390/nu8110729 PMC5133113

[32] Cheng Z , Tu J, Zhang H, zhang Y, Zhou B. Urolithin A attenuates renal fibrosis by inhibiting TGF-β1/Smad and MAPK signaling pathways. J Funct Foods 2021;83:104547.

[33] Chen P , Pei J, Wang X, Tai S, Tang L, Hu X. Gut bacterial metabolite Urolithin A inhibits myocardial fibrosis through activation of Nrf2 pathway *in vitro* and *in vivo*. Mol Med 2022;28:19. 35135471 10.1186/s10020-022-00444-1 PMC8822684

[34] Yang Q , Guo N, Zhou Y, Chen J, Wei Q, Han M. The role of tumor-associated macrophages (TAMs) in tumor progression and relevant advance in targeted therapy. Acta Pharm Sin B 2020;10:2156–70. 33304783 10.1016/j.apsb.2020.04.004 PMC7714989

[35] Kravtsov DS , Erbe AK, Sondel PM, Rakhmilevich AL. Roles of CD4+ T cells as mediators of antitumor immunity. Front Immunol 2022;13:972021. 36159781 10.3389/fimmu.2022.972021 PMC9500154

[36] Denk D , Petrocelli V, Conche C, Drachsler M, Ziegler PK, Braun A, . Expansion of T memory stem cells with superior anti-tumor immunity by Urolithin A-induced mitophagy. Immunity 2022;55:2059–73. 36351375 10.1016/j.immuni.2022.09.014

[37] Budimir N , Thomas GD, Dolina JS, Salek-Ardakani S. Reversing T-cell exhaustion in cancer: lessons learned from PD-1/PD-L1 immune checkpoint blockade. Cancer Immunol Res 2022;10:146–53. 34937730 10.1158/2326-6066.CIR-21-0515

[38] Peng Q , Qiu X, Zhang Z, Zhang S, Zhang Y, Liang Y, . PD-L1 on dendritic cells attenuates T cell activation and regulates response to immune checkpoint blockade. Nat Commun 2020;11:4835. 32973173 10.1038/s41467-020-18570-x PMC7518441

[39] Pu Y , Ji Q. Tumor-associated macrophages regulate PD-1/PD-L1 immunosuppression. Front Immunol 2022;13:874589. 35592338 10.3389/fimmu.2022.874589 PMC9110638

[40] Liu Y , Zugazagoitia J, Ahmed FS, Henick BS, Gettinger SN, Herbst RS, . Immune Cell PD-L1 colocalizes with macrophages and is associated with outcome in PD-1 pathway blockade therapy. Clin Cancer Res 2020;26:970–7. 31615933 10.1158/1078-0432.CCR-19-1040 PMC7024671

[41] Ho TTB , Nasti A, Seki A, Komura T, Inui H, Kozaka T, . Combination of gemcitabine and anti-PD-1 antibody enhances the anticancer effect of M1 macrophages and the Th1 response in a murine model of pancreatic cancer liver metastasis. J Immunother Cancer 2020;8:e001367. 33188035 10.1136/jitc-2020-001367 PMC7668383

