# Supplementary material for: Remodeling of Stromal Immune Microenvironment by Urolithin A Improves Survival with Immune Checkpoint Blockade in Pancreatic Cancer
Source: Cancer Res Commun. 2023 Jul 12;3(7):1224–36. doi: 10.1158/2767-9764.CRC-22-0329 (PMC10337606; doi:10.1158/2767-9764.CRC-22-0329)
Supplement: Figure S1 — Raw uncropped images of Western blot membranes for Figs.1e and 3d. [file crc-22-0329-s01.pdf]

Figure 1e

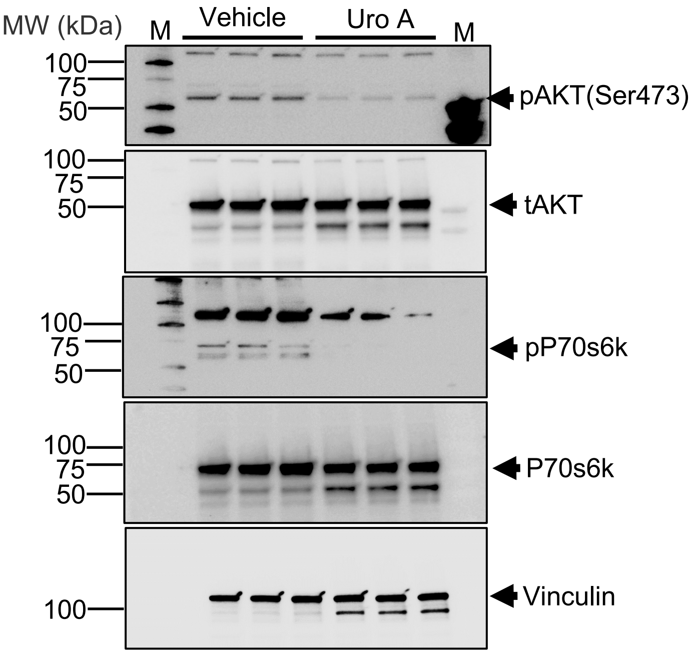

Figure 3d

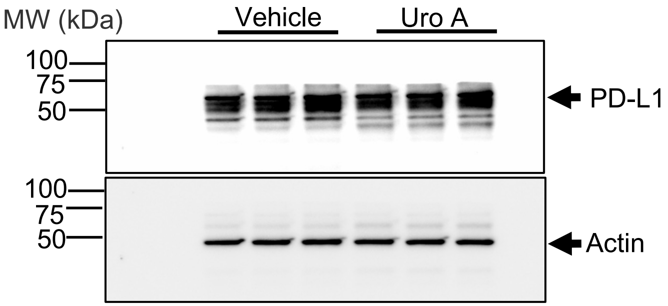

Supplementary Figure S1. Raw uncropped images of Western blot membranes for Figs. 1e and 3d.
