# Supplementary figures and images for: Remodeling of Stromal Immune Microenvironment by Urolithin A Improves Survival with Immune Checkpoint Blockade in Pancreatic Cancer
Source: Cancer Res Commun. 2023 Jul 12;3(7):1224–36. doi: 10.1158/2767-9764.CRC-22-0329 (PMC10337606; doi:10.1158/2767-9764.CRC-22-0329)

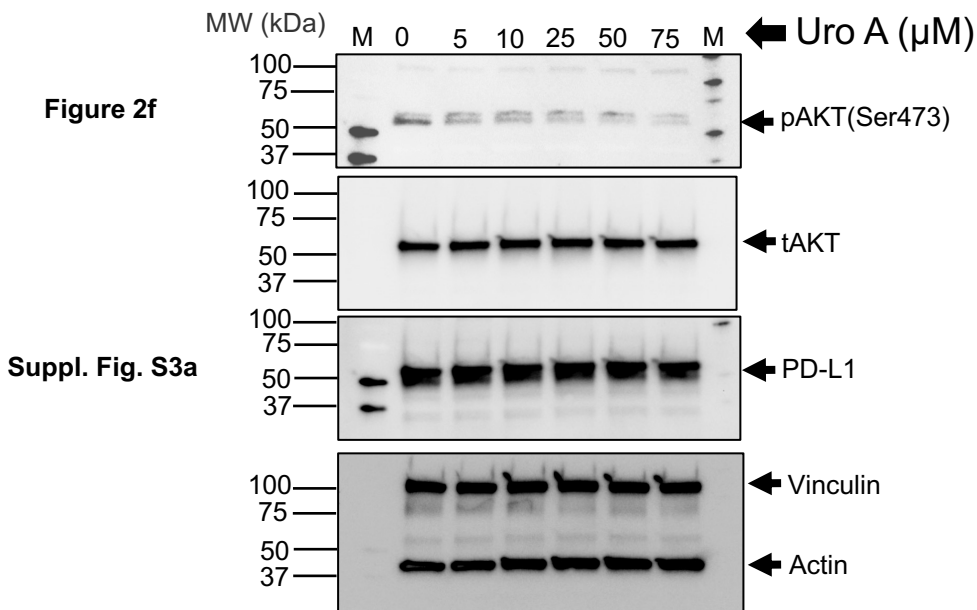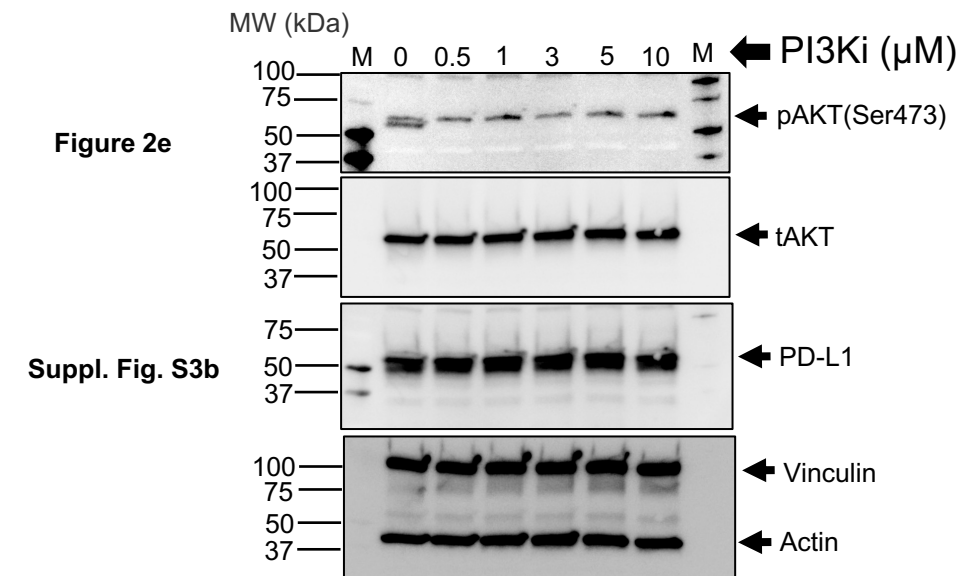

**Supplementary Figure S2.** Raw uncropped images of Western blot membranes for Figs. 2e, 2f, S3a and S3b.

Supplement: Figure S2 — Raw uncropped images of Western blot membranes for Figs. 2e, 2f, S3a and S3b. [file crc-22-0329-s02.pdf]
