# Supplementary material for: Remodeling of Stromal Immune Microenvironment by Urolithin A Improves Survival with Immune Checkpoint Blockade in Pancreatic Cancer
Source: Cancer Res Commun. 2023 Jul 12;3(7):1224–36. doi: 10.1158/2767-9764.CRC-22-0329 (PMC10337606; doi:10.1158/2767-9764.CRC-22-0329)
Supplement: Figure S3 — Gating strategies used for flow cytometry analysis. [file crc-22-0329-s03.pdf]

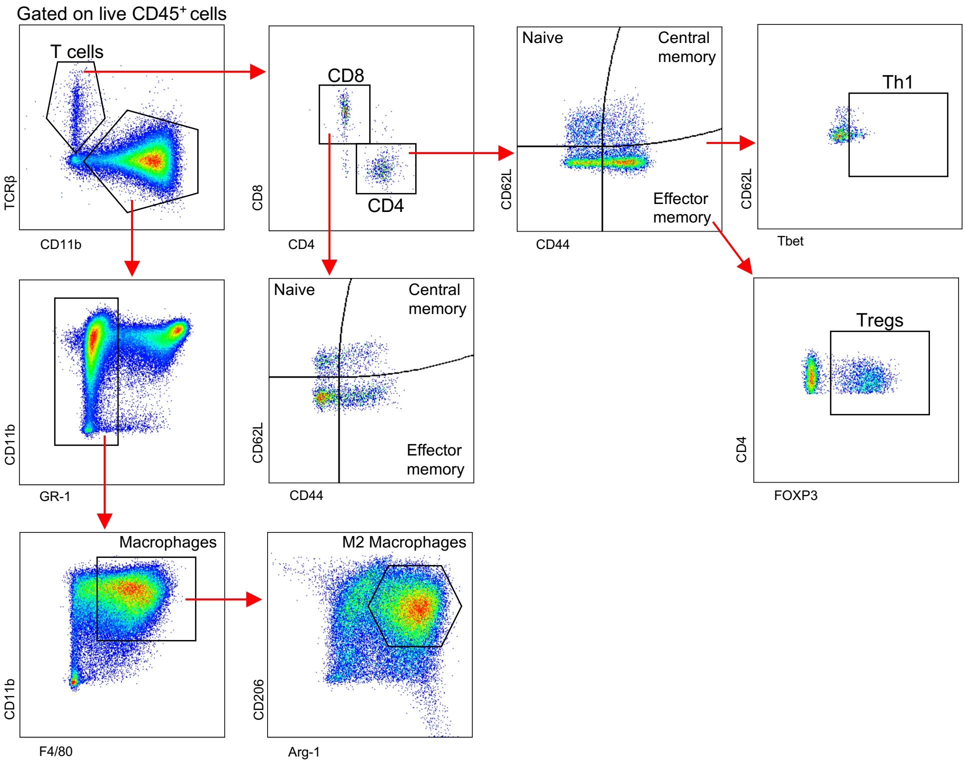

Supplementary Figure S3. Gating strategies used for flow cytometry analysis. Representative dot plots depict gating strategies used for intratumoral macrophages and T-cell analysis in flow cytometry experiments in PKT mice across all treatment cohorts.
