# Supplementary material for: Remodeling of Stromal Immune Microenvironment by Urolithin A Improves Survival with Immune Checkpoint Blockade in Pancreatic Cancer
Source: Cancer Res Commun. 2023 Jul 12;3(7):1224–36. doi: 10.1158/2767-9764.CRC-22-0329 (PMC10337606; doi:10.1158/2767-9764.CRC-22-0329)
Supplement: Figure S4 — Tumor weight, mouse weight and cytokine profiling of Uro A treatment in PKT mice. [file crc-22-0329-s04.pdf]

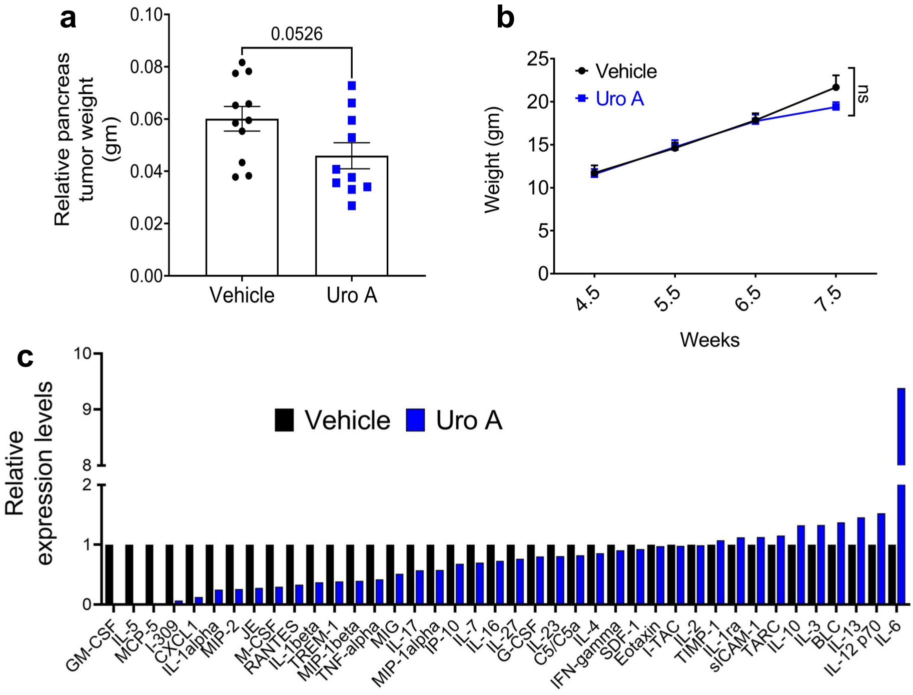

Supplementary Figure S4. Tumor weight, mouse weight and cytokine profiling of Uro A treatment in PKT mice. (a) Relative pancreas tumor weight normalized to body weight in PKT mice treated with Uro A or vehicle. **(b)** Body weights of PKT mice were recorded weekly during the treatment regimen across all groups. **(c)** Profiling of intratumoral immunomodulatory cytokines in Uro A or vehicle-treated PKT mice. ns: non-significant.
