# Supplementary material for: Remodeling of Stromal Immune Microenvironment by Urolithin A Improves Survival with Immune Checkpoint Blockade in Pancreatic Cancer
Source: Cancer Res Commun. 2023 Jul 12;3(7):1224–36. doi: 10.1158/2767-9764.CRC-22-0329 (PMC10337606; doi:10.1158/2767-9764.CRC-22-0329)
Supplement: Figure S5 — Cytokine profiling of PKT tumors and KPC cells treated with PI3Ki or Uro A. [file crc-22-0329-s05.pdf]

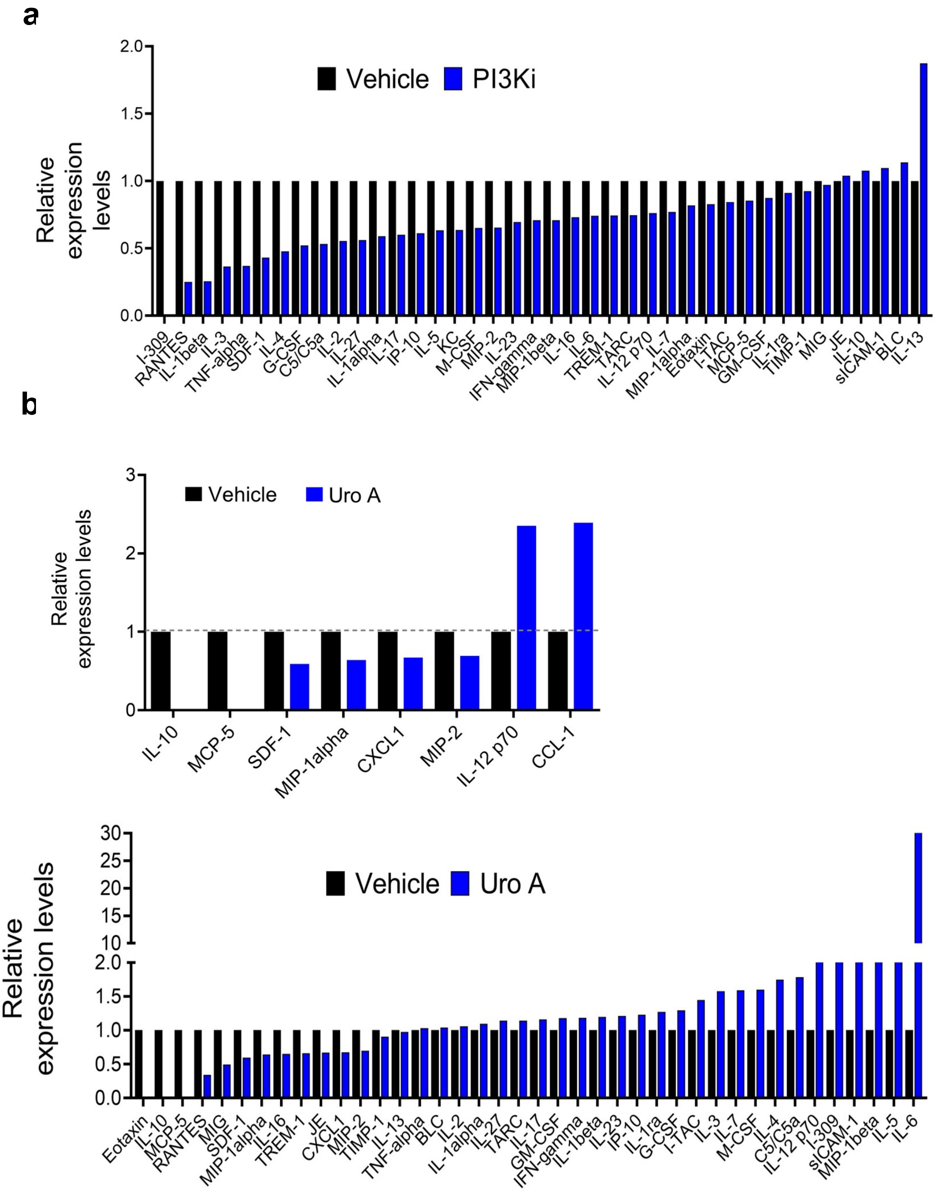

Supplementary Figure S5. Cytokine profiling of PKT tumors and KPC cells treated with PI3Ki or Uro A. (a) Profiling of intratumoral immunomodulatory cytokines in Omipalisib (PI3Ki) treated or vehicle-treated PKT mice. **(b)** Profiling of immunomodulatory secretome in the conditioned media of KPC PDAC tumor cells harvested after Uro A (25μM) treatment for 3 hours.
