# Supplementary material for: Remodeling of Stromal Immune Microenvironment by Urolithin A Improves Survival with Immune Checkpoint Blockade in Pancreatic Cancer
Source: Cancer Res Commun. 2023 Jul 12;3(7):1224–36. doi: 10.1158/2767-9764.CRC-22-0329 (PMC10337606; doi:10.1158/2767-9764.CRC-22-0329)
Supplement: Figure S6 — PD-L1 expression in Uro A or PI3Ki treated KPC cells. [file crc-22-0329-s06.pdf]

a

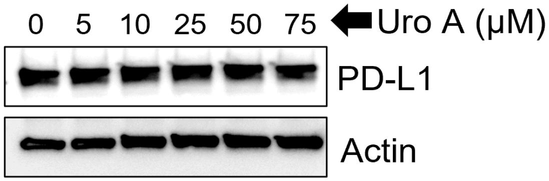

b

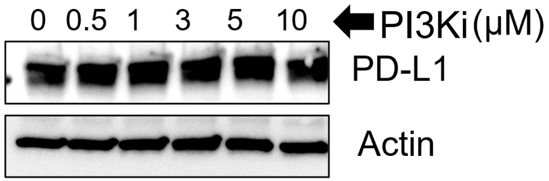

Supplementary Figure S6. PD-L1 expression in Uro A or PI3Ki treated KPC cells. (a) and (b) Western blot analysis of KPC PDAC cells demonstrating PD-L1 expression with increasing concentration of Uro A (5-75μM) and PI3Ki (0.5-10μM).
