# Supplementary material for: Remodeling of Stromal Immune Microenvironment by Urolithin A Improves Survival with Immune Checkpoint Blockade in Pancreatic Cancer
Source: Cancer Res Commun. 2023 Jul 12;3(7):1224–36. doi: 10.1158/2767-9764.CRC-22-0329 (PMC10337606; doi:10.1158/2767-9764.CRC-22-0329)
Supplement: Figure S7 — Recorded body weights of PKT mice treated with Uro A, aPD-1 and Uro A+aPD-1. [file crc-22-0329-s07.pdf]

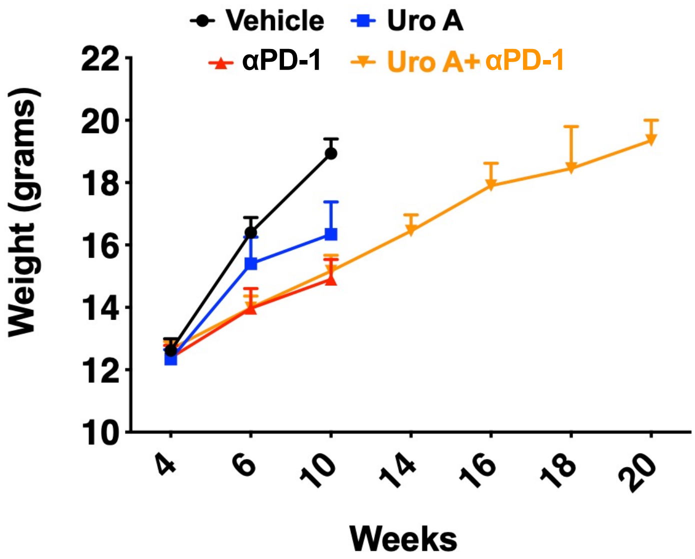

Supplementary Figure S7. Recorded body weights of PKT mice treated with Uro A, aPD-1 and Uro A+aPD-1. PKT mice body weights were recorded weekly while mice received vehicle control, Uro A (20 mg/kg/daily), anti-PD-1 antibody (200μg/mouse), or a combination of Uro A with anti-PD-1 antibody. ns: non-significant.
