# Supplementary material for: Remodeling of Stromal Immune Microenvironment by Urolithin A Improves Survival with Immune Checkpoint Blockade in Pancreatic Cancer
Source: Cancer Res Commun. 2023 Jul 12;3(7):1224–36. doi: 10.1158/2767-9764.CRC-22-0329 (PMC10337606; doi:10.1158/2767-9764.CRC-22-0329)
Supplement: Supplementary Tables S1-S2 — Primary antibodies for flow cytometry, immunohistochemistry and Western blot analysis. [file crc-22-0329-s08.docx]

**SUPPLEMENTARY TABLES**

**Supplementary Table S1.** Primary antibodies for histological and Western blot analysis.

| **Primary Antibodies** | **Supplier** | **Species** | **Catalogue number** |
| --- | --- | --- | --- |
| pAKT | Cell Signaling | Rabbit | 4060 |
| pP70S6K | Cell Signaling | Rabbit | 9204 |
| tAKT | Cell Signaling | Rabbit | 4685 |
| tP70S6K | Cell Signaling | Rabbit | 34475 |
| PD-L1 | Abcam | Rabbit | 213480 |
| Collagen-I | Abcam | Rabbit | ab5694 |
| Vinculin | Cell Signaling | Rabbit | 13901 |
| Actin | Cell Signaling | Rabbit | 58169 |

**Supplementary Table S2.** Primary antibodies for flow cytometry analysis.

| **Primary Antibodies** | **Fluorophore** | **Supplier** | **Species** | **Catalogue number** |
| --- | --- | --- | --- | --- |
| CD45 | BUV-805 | Biolegend | Rat | 103108 |
| TCR-β | PerCPCy5.5 | Biolegend | Rat | 100218 |
| CD4 | BV-785 | Biolegend | Rat | 100453 |
| CD8 | BV-605 | Biolegend | Rat | 100744 |
| PD-1 | PE-Cy7 | Biolegend | Rat | 135216 |
| PD-L1 | BV-421 | Biolegend | Rat | 124315 |
| CD44 | BUV-805 | Biolegend | Rat | 741921 |
| CD62L | BUV-563 | BD | Rat | 741230 |
| TBET | Kiravia Blue | Biolegend | Mouse | 644838 |
| FOXP3 | PE | Biolegend | Rat | 126404 |
| CD11b | BV-750 | Biolegend | Rat | 101267 |
| F4/80 | AF-488 | Biolegend | Rat | 123120 |
| Arg-1 | APC | Invitrogen | Rat | 17-3697-82 |
| CD206 | BV-650 | Biolegend | Rat | 141723 |
| MHC-II | APC Fire-750 | Biolegend | Mouse | 116424 |
